# Mesenchymal stem cells from biology to therapy

**DOI:** 10.1042/ETLS20200303

**Published:** 2021-08-06

**Authors:** David Kuntin, Paul Genever

**Affiliations:** Department of Biology, University of York, York YO10 5DD, U.K.

**Keywords:** cell therapy, extracellular vesicle, mesenchymal stem cell, mesenchymal stromal cells, regenerative medicine, tissue engineering

## Abstract

Mesenchymal stem cells are as fascinating as they are enigmatic. They appear capable of performing a wide array of functions that cross skeletal biology, immunology and haematology. As therapeutics, mesenchymal stem cells or even just their secreted products may be used to regenerate tissue lost through injury or disease and suppress damaging immune reactions. However, these cells lack unique markers and are hard to identify and isolate as pure cell populations. They are often grown in laboratories using basic and undefined culture conditions. We cannot even agree on their name. While mesenchymal stem cells may lack the developmental understanding and defined differentiation hierarchies of their more illustrious stem cell cousins, they offer a compelling scientific challenge. In depth understanding of mesenchymal stem cell biology will enable us to exploit fully one of the most clinically valuable cell sources.

## MSC discovery and biology

In 1966, Friedenstein et al. [1] demonstrated that cells derived from mouse bone marrow, as well as other blood-forming organs, contain a subpopulation of stem-like cells that give rise to bone cell precursors. In this seminal paper, these cells were named osteogenic stem cells, although with further study, Friedenstein realised their greater potential to differentiate into fat and cartilage precursors, too [2,3]. In 1991, Caplan [4] coined the term ‘mesenchymal stem cell’ and the abbreviation ‘MSC’, which has since remained the most commonly used moniker. The notion that MSCs have trilineage potential, i.e. the capacity to differentiate into bone, cartilage and fat cells, was developed further in the 1999 report by Pittenger et al. [5], where bone marrow cells isolated from iliac crest aspirates were shown to differentiate into these lineages *in vitro* with the addition of differentiation-specific stimuli. Many further studies have since reproduced these methods and built on them.

Expanding research activity and evolution of the field made clear the more complex nature of these cells and that technically limited isolation techniques often failed to select a homogeneous *stem cell* population. It was thought that the name should reflect this, with proposed MSC expansions including ‘multipotent stromal cells’, ‘mesenchymal stromal cells’ [6] and even ‘medicinal signalling cells’ [7]. Some of this nomenclature actually refers to specific subpopulations of cells isolated from tissues by plastic adherence, while others are an attempt at broadening the term. In either case, it can confuse discourse and conflate smaller, more specialised subpopulations, with the overall, heterogeneous cell population. Some authors use the term ‘skeletal stem cell (SSC)’, recognising that a stem cell population exists in adult bone marrow, capable of forming bone, cartilage, fat, and haematopoietic supporting tissue [8,9]. The SSC term also removes reference to embryonic mesenchyme, which implies the capacity to differentiate in all mesenchyme-derived cells and tissues including blood cells. The naming of these cells continues to be debated [10]. For the remainder of this review, the term ‘mesenchymal stromal cells’ will be used for MSCs, to refer to the broader population of cells, and to acknowledge their heterogeneity and the fact that not all plastic-adherent cells isolated from sources such as bone marrow and fat have multipotent differentiation capability. This view is in line with the International Society for Cellular Therapy (ISCT) position paper first published in 2005 [6], where MSCs were defined to be plastic-adherent cells, derived from several tissues, such as bone marrow, umbilical cord or fat, with the potential to differentiate into bone, cartilage, and fat cells. They should also express the cell surface proteins CD105, CD73 and CD90, and lack CD45, CD34, CD14 or CD11b, CD79 or CD19 and HLA-DR [11], though it is important to note that this statement specifies that these are minimal criteria and a starting point for further study. This publication is often referred to in MSC-related literature to assure the reader that the MSCs used in the study in question met the ‘ISCT criteria’, though there are pitfalls with this approach, which we discuss later in this review. The ISCT criteria were later expanded [12] to recommend the inclusion of tissue source when referring to particular MSC populations used in experimental work, alongside a robust body of evidence clarifying whether *stem-*like cells or *stromal-*like cells are being presented, with emphasis on the fact that mesenchymal *stem* cells represent self-renewing, multipotent cells, while mesenchymal *stromal* cells describe bulk, unfractionated cells.

While most of this work attempted to define an *in vitro* expanded MSC population, there has been some progress in identifying the *in vivo* location of MSCs, or ‘niche’, focusing mainly on murine skeletal tissues. The niche is a specialised tissue microenvironment that houses and regulates the function of an adult stem cell [13,14]. Stem and progenitor cells that give rise to osteogenic and chondrogenic lineages have been identified primarily around blood vessels in bone marrow [15–19] and more recently, the outer bone surface [20] and the growth plate of cartilage [21–23]. Much of the work on bone marrow MSCs has analysed stromal cells as *in vivo* regulators of the haematopoietic stem cell (HSC) niche [18,24]. Through these and related studies, MSCs have been identified by their production of HSC-niche regulatory factors, such as CXCL12 and stem cell factor (SCF) and the expression of leptin receptor (LEPR), Nestin and CD146, amongst others [17,19,25,26]. Using in-depth gene profiling techniques, up to 17 different subtypes of related stromal cells have been identified [27]. A clear picture of the *in vivo* ‘MSC map’ is still developing, which will be aided by the emergence of advanced spatial profiling techniques; see Dolgalev and Tikhonova [28] for a recent extensive review. Further studies of MSCs *in vivo* using human tissues are needed, particularly due to the differences in postnatal mouse and human long bone development [29]. In situ analyses of MSCs in different tissues will also provide better biological understanding and more appropriate terminology linked to tissue-specific subtypes. Effects of factors such as oxygen tension [30] and cell–cell interactions will be of particular interest, as this could shed light on the nature of the *in vivo* MSC environment, which may inform bioengineering approaches to maintaining MSCs *ex vivo* in as natural a state as possible [30,31].

**Figure 1.**
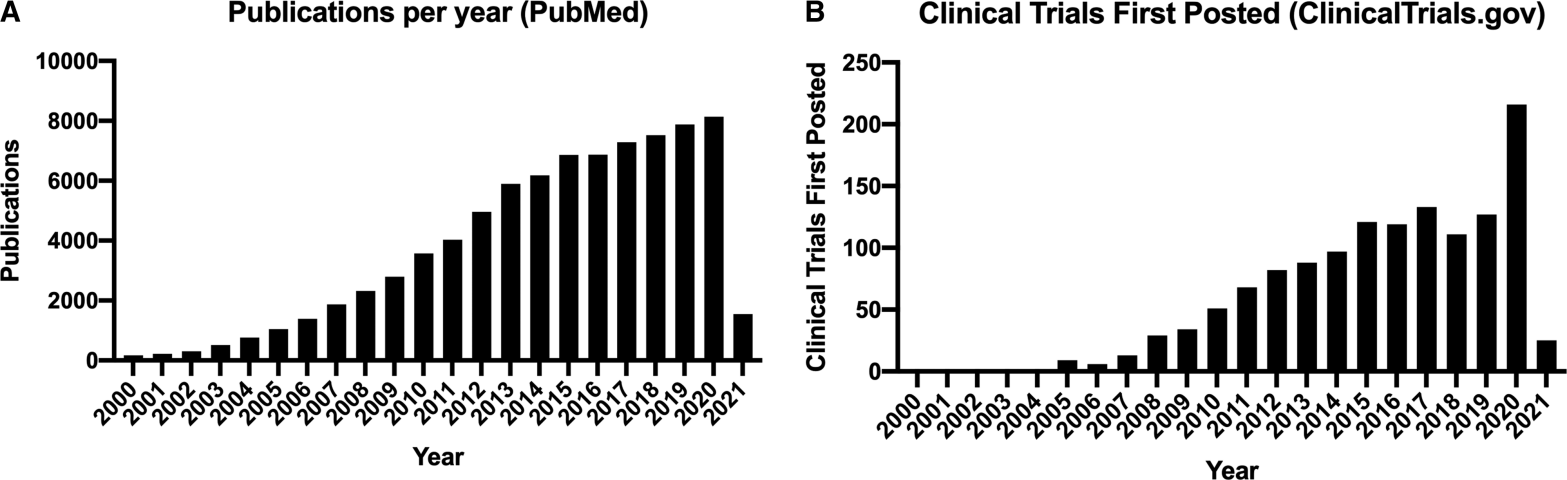
Activity in the research and clinical trial fields using MSCs. (**A**) Number of publications listed on PubMed database (search term: ‘mesenchymal stem cells’ OR ‘mesenchymal stromal cells’) by year from 2000. (**B**) Clinical trials first posted (search term on ClinicalTrials.gov: ‘mesenchymal stem cells’ OR ‘mesenchymal stromal cells’) by year from 2000.

The issue of MSC identity is complicated further as MSC-*like* cells have been isolated from a myriad of tissues, though most commonly from bone marrow and adipose tissue from adults. The umbilical cord and placenta are also accessible sources of MSCs, as these are often considered medical waste. To achieve relevant cell numbers, MSCs are usually culture expanded for both research and clinical applications, which is an easily reproducible procedure in the laboratory. Simple MSC isolation and expansion procedures and their clinically appealing regenerative potential underlie the steady increase in the number of publications and clinical trials using MSCs, especially since the year 2000 (Figure 1).

However, it is important to bear in mind that cultured MSCs differ substantially from their native physiological state, due to the vastly different environmental conditions. Culture expansion removes tissue-specific biological cues of the niche, including the presence of different cell types, extracellular matrix (ECM) components and oxygen gradients, and may disguise true *in vivo* function. The question of whether the therapeutic potential we observe in the laboratory is a product of the process these cells undergo when they are isolated from their tissues and expanded, or in fact reflects their natural function in the organism is a matter of further research. Indeed, if considering the bone marrow niche as an example, which appears varied and complex based on the evidence from single cell and spatial profiling studies described above, tissue culture conditions differ substantially. The presence of animal serum allows for colony formation and expansion of cells, with abundant nutrition and stimulation to remain in culture for extended periods of time. It is, however, understood that within the bone marrow, MSCs maintain their stem-like properties, at least in part, through specific cell–cell interactions. These are comparatively less abundant once the cells are introduced into a culture vessel. The serum contains ECM proteins, such as fibronectin and collagens, which prompt the formation of extensive cytoskeletal networks as cells attach and spread on a rigid, flattened surface. The cells interact with their substrate through integrin-mediated focal adhesions, which is thought to influence their fate [32,33].

Integrin-based interactions are also involved in directing MSC function through substrate stiffness. On stiff surfaces, MSCs were shown to exhibit a tendency for osteogenic differentiation, based on alkaline phosphatase activity, osteogenic gene marker expression, and calcium staining. In addition to increased expression of several integrins, increased activation of downstream signalling events, for example via focal adhesion kinase (FAK), phosphorylated extracellular signalling regulated kinase (pERK), phosphorylated Akt, glycogen synthase kinase 3β (GSK3β), and β-catenin, have been observed, indicating a complex mechanotransduction cascade mediating the effect of substrate stiffness on cell fate [34–37]. Soft surfaces, on the other hand, have been shown to maintain MSC self-renewal capacity and appear to promote adipogenic differentiation [38,39]. This property of soft hydrogels, thought to be via Yes-associated protein-1 (YAP) signalling, is being investigated as a strategy for maintaining MSC surface marker expression patterns associated with their regenerative properties, which are lost over time in culture [40]. In general, material stiffnesses mimicking those of certain tissues tends to condition MSCs to adapt to this and induces gene expression patterns consistent with corresponding MSC niches (reviewed in [41]), explaining to some extent the propensity for certain lineages on particular substrates.

While *in vitro* analyses may offer only an interpretation of the true biological nature of MSCs, it is clear from this work that MSCs have substantial clinical potential and that there are opportunities to use these cells as therapeutics in a broad range of applications.

## MSC therapeutic approaches

**Figure 2.**
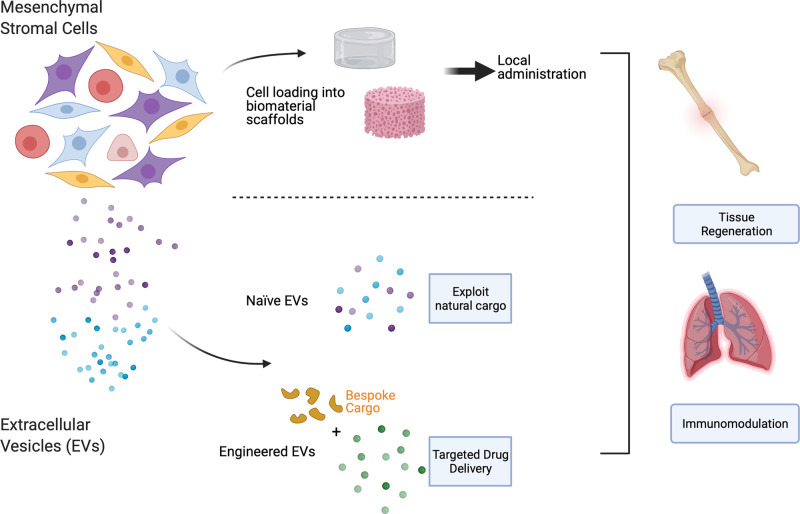
Approaches to therapeutic exploitation of MSCs and their products. MSCs can be applied by direct injection of cell suspensions or seeded onto biomaterial scaffolds as adhesion sites for local administration. MSC-derived EVs can be used in their naïve, unaltered state or engineered to carry specific cargos and/or cell-targeting motifs. Both modalities are applicable in tissue regeneration or immunomodulatory therapies.

Autologous and allogeneic sources of MSCs have been used as cell therapies for many years and form the vast majority of clinical trials identified in Figure 1B. Recently, interest in the use of MSC-derived bioactive products — those secreted by MSCs into the extracellular environment — has increased markedly. We will cover both these approaches under ‘Cell-based therapies’ and ‘Cell-derived therapies’ below (see also Figure 2).

## Cell-based therapies

Therapeutic approaches exploiting MSC biology focus on their ability to differentiate into new tissues and act as modulators of the immune system. Early work investigating MSCs for their therapeutic utility demonstrated that MSCs have certain immunomodulatory characteristics that allow them to persist in a xenogeneic environment. To demonstrate this, Liechty et al. [42] introduced human MSCs into sheep foetuses both before and after the foetuses were expected to develop immune-competence. The cells successfully engrafted in both cases and integrated into the developing tissues, undergoing site-specific differentiation. This immunomodulatory capability, coupled with their tissue-forming capacity, provides MSCs with their unique therapeutic value. Clinical targets for MSC therapies include inflammatory indications such as graft versus host disease (GvHD) [43] and rheumatoid arthritis [44], as well as for the purposes of tissue regeneration, such as osteogenesis imperfecta [45,46] and large bone defects [47,48]. In our recent analysis of all published clinical trials (2009–2019) using MSCs, we identified 35 different indications, most commonly those affecting the nervous, cardiovascular and musculoskeletal systems [49].

Many MSC-based interventions rely on MSCs homing to the target site following systemic injection. MSC homing is thought to be cytokine and surface antigen regulated, and refers to the idea that MSCs, when injected systemically into the bloodstream or administered locally, preferentially migrate toward sites of injury [50]. While systemic administration has its benefits, such as being the least invasive means of delivering MSCs, it has been shown that homing to the desired tissue can be very inefficient [51–53], resulting in low levels of engraftment, mainly due to entrapment in the lung microvasculature [54]. Many strategies are being investigated to improve MSC homing, focusing on making patients more receptive to MSCs [55,56] or engineering the MSCs to avoid problems related to patient responses to systemically administered cells [57,58]. It should be noted that in order to translate this approach into viable therapies, scale-up must be addressed. MSC doses are generally in the region of 1–2 million cells/Kg [59], which poses a particular challenge related to culture-associated loss of specific MSC markers used as critical quality attributes in the manufacture of MSC therapeutics [60].

MSCs can be administered in a more targeted manner by local administration using scaffolds (Figure 2). To address the issue of low engraftment, a biomaterial scaffold is often used to provide a three-dimensional (3D) structure with a high surface area for cell adhesion, especially when large areas of damaged tissue need to be replaced and/or mechanical strength is required, for example in bone and cartilage replacements, where much of the activity in this area has focused. The aim of this approach is to mimic the tissue microenvironment. Biomimetic scaffolds can range from simply imitating the stiffness or general architecture of the tissue in question, to being doped with specific growth factors and coated with matrix proteins to coax MSCs into a particular lineage. For example, this concept can be applied to critical size bone defects, where a physical structure is required to administer MSCs. Persson and colleagues describe an 80 : 20 mixture of polylactic acid (PLA) and hydroxyapatite (HA), which was used to fabricate a woven scaffold with specific porosity and pore size. These scaffolds were shown to promote MSC proliferation, as well as supporting osteoblastic differentiation and mineralised bone matrix formation in critical size defects [61]. Scaffolds can also be more complex composites, and even can be personalised, by combining state-of-the-art engineering techniques with current knowledge of MSC biology. An example of this was demonstrated by Kuss et al., where a 3D-printed polycaprolactone (PCL)/HA composite scaffold was constructed, then coated with a complex, cell-laden hydrogel, with the aim of improving vascularisation. The hydrogel contained a mixture of adipose-derived MSCs and human umbilical vein endothelial cells. This essentially prevascularized the construct, demonstrating the possibility of creating an already vascularised scaffold, made to fit unique anatomical structures [62]. For further information on the use of 3D scaffolds for MSC delivery, tissue regeneration, directing cell function, immunomodulation and genetic modification, please refer to recent reviews [63–66].

These studies are promising steps toward regenerative solutions to tissue repair by effectively engaging in multidisciplinary research to advance our understanding of how materials integrate into and interact with tissues to achieve optimal regeneration.

## Cell-derived therapies

While cell-based therapies are proving encouraging, there has been growing interest in the use of cell-derived material for therapeutic purposes. Bioactive factors produced by cells, extracellular vesicles (EVs) in particular, can reflect the functions of the cell from which they originate. EVs are nanoscale, membranous particles secreted from cells, containing diverse cargo including nucleic acids, such as miRNA, and proteins. It has been shown that EVs mediate cell-to-cell communication by shuttling biomolecules to influence the microenvironment [67–70]. Given that the function of EVs is to act as signalling particles for surrounding cells, it follows that the signals carried by the EVs could be harnessed to deliver desirable biological factors to target cells, affording them innate therapeutic utility (Figure 2). The EV field has grown hugely in recent years and several recent reviews describe in more detail EV biogenesis, function and clinical possibilities [71–73].

EVs are also being viewed as delivery vehicles (see Figure 2). Engineering EVs to transport specific cargo is an attractive prospect as they carry surface molecules which could aid in targeted delivery [74,75]. EVs can be loaded with proteins, nucleic acids, or small molecules by either modifying the producing cell or by directly loading the EVs, making this a versatile platform for drug delivery [76].

MSCs seem to be a particularly good source of EVs. Studies have shown that EVs derived from MSCs are more stable than those derived from other cell types [77], and the capabilities that MSCs exhibit in terms of their differentiation and immunomodulation potential leads to naturally clinically potent EVs. It could also be more efficient to use EVs from MSCs over the MSCs themselves, as EVs are produced constantly, so could be harvested as MSCs are expanded. Cell therapy on the other hand, would generally require cells to be expanded up to the point where they are used in that therapy. EV production can be assisted by MSC immortalisation, which has already been demonstrated by some groups [78–80], which gives rise to an inexhaustible source of therapeutically useful MSC-EVs, effectively eliminating batch variability; a problem inherent to the use of primary donor cells.

While there are currently no approved treatments available using EVs, there is an increasing body of published data pointing toward the clinical utility of EVs for many indications. The function of EVs in fracture healing, for example, was demonstrated in CD9 knockout mice, which were shown to have impaired EV biogenesis [81], as well as lowered rates of bone repair, as exhibited by retardation of callous formation [82], compared with wild type. Furuta and colleagues showed that this retardation was rescued by injection of EVs isolated from the conditioned medium of bone marrow-derived MSCs, but not from EV-free conditioned medium. Work by Qin et al. [83] further demonstrated that EVs from bone marrow-derived MSCs could enhance bone formation in calvarial defects of Sprague Dawley rats, with miR-196a identified as critical in regulating osteoblastic differentiation and osteogenic gene expression.

There has also been a lot of interest in the use of EVs for the attenuation of the after-effects of COVID-19, caused by the severe acute respiratory syndrome coronavirus 2 (SARS-CoV-2). The mechanism(s) of action is yet to be fully understood but revolve around dampening the aggravated inflammatory effects of the respiratory system and repairing tissue damage. It has been suggested in work completed before the onset of this novel coronavirus that inflammation in the lungs could be controlled using MSC-derived EVs by immune cell modulation [84], the notion of which has been put under more intense investigation as a result of the COVID-19 pandemic, though appropriately controlled trials are required [85].

## Current challenges

It is an accepted truth that cultured cells termed ‘MSCs’ are vastly heterogeneous, as MSCs differ depending on donor/tissue source, isolation/culture technique, and inherent heterogeneity. With the ISCT position statement in 2006, there has been an attempt at harmonisation across groups, which is a positive step forward, but the lack of standardised criteria for the identification and classification of MSC subpopulations presents a substantial obstacle to the development of MSC therapies. More work is required to further our understanding of MSC identity to move the field forward effectively.

A further challenge in the current approach to MSC therapy is the reliance on donor-derived cells for MSC-based therapy scale-up. Whether relying on MSCs themselves to deliver a therapeutic effect, or harvesting MSC-derived factors, MSCs will have to be culture expanded *ex vivo* to produce clinically usable doses. Using donor-derived cells, which will differ from donor-to-donor, introduces an extra quality control step into production, where there is the potential for many batches to be rejected. Additionally, there is an overwhelming reliance on animal-derived culture additives to produce the quantity of cells required for therapeutic use, which is both ethically and scientifically challenging. The most commonly used additive, foetal bovine serum (FBS), is unsustainably sourced, with the global demand of FBS increasing and the supply struggling to keep up [86,87]. FBS is a complex, undefined mixture, suffering from batch variability. Sources of variability cause major problems in the development of therapeutics, where consistency is key to overcoming regulatory burdens and successfully scaling up production. Xeno-free medium solutions are available, but there is a tendency for life science companies to develop proprietary formulations to protect commercial interests. As far as the research community is concerned, commercially available media are still undefined while being very costly. A chemically defined, non-proprietary medium would aid standardisation across MSC research groups and assist the development and manufacture of MSCs, and their secreted products, for clinical use.

The field of EV therapeutics is an emerging one and we still find ourselves in the early stages of developing and determining a gold standard set of processes by which EVs can be produced, harvested, isolated, and characterised. The problems are similar to those plaguing MSCs currently, as EVs are broadly characterised based on their size and how they were formed, often using marker expression and imaging as readouts. One example of how this problem becomes evident is the fact that the method by which EVs are isolated generally determines the identity of the resulting EV preparation. Currently, the most commonly used isolation technique involves differential ultracentrifugation, which is effective, but fairly crude and time-consuming. To address this, many researchers have developed other isolation techniques and the EV size distribution and yield, quality, and function differs between techniques [88,89].

The International Society for Extracellular Vesicles (ISEV) has published a position statement, similar to the ISCT in 2006, outlining a list of suggested protocols and recommendations on specific criteria to be reported in order to aid in the advancement of the field as a whole with a unified vocabulary. These guidelines also point out that it is an evolving document, and that new technologies are arising regularly, and that the aim is to enhance communication between researchers [90]. Communication is key.

With regard to EV functionality and their use to address the COVID-19 pandemic, ISCT and ISEV issued a joint statement encouraging investigations into MSC-derived EVs, as well as possibly other cell sources, as treatments for COVID-19, recognising their potential in this area, but stressing that they do not currently endorse their use without sufficient evidence of their safety and efficacy, alongside several more provisions related to clinical studies, manufacture, and regulation [85]. EVs are a rapidly growing, exciting field of research but careful consideration needs to be given to their mechanisms of action to ensure that these are used in a targeted manner, for maximal efficacy. Our currently limited understanding of factors underlying COVID-19 complications, as well as the complex mechanisms of action of EV interventions are an obstacle to good clinical trial design [91]. Further work into understanding the very nature of EVs is required to effectively design EV therapeutics.

## Conclusions and future directions

MSCs are an exciting cell population. A vast amount of work is attempting to translate MSCs and related technologies into viable therapeutics for an enormous range of applications. In this review, we touched on some of the key target tissues, bone in particular, but the research is being developed in many more areas, including nerve, heart, cartilage, liver, kidney and, as we discussed above, virally induced inflammatory lung disorders. There are new and improved delivery methods in the pipeline, such as hydrogels for cells [92] and intranasal aerosols for EVs [93]. The emergence of EVs as a therapeutic modality has opened the doors to cell-free regenerative medicines, with great versatility and utility. That is not to say that cell therapies will be surpassed by EVs, but EVs are a powerful offshoot of traditional cell therapies with the potential to disrupt the regenerative medicine space. It is important to remember that while excitement continues to grow for MSC-based therapies, clinical development must always follow scientific understanding. There is much we still need to do in order to decipher the enigmatic MSC.

## Summary

- Mesenchymal *stem* cells are frequently studied for research and clinical use as heterogeneous cell populations, giving rise to the term mesenchymal *stromal* cells (MSCs).
- MSCs have wide-ranging therapeutic applications but aspects of MSC biology require further work in order to maximise their potential.
- MSC-derived EVs are an emerging therapeutic modality.
- A harmonised approach to defining and analysing MSCs and MSC-EVs is essential for effective communication within the research community to facilitate progression within the field.

## Open Access Statement

Open access for this article was enabled by the participation of University of York in an all-inclusive *Read & Publish* pilot with Portland Press and the Biochemical Society under a transformative agreement with JISC.

## Abbreviations

ECM: extracellular matrix
FBS: foetal bovine serum
HA: hydroxyapatite
HSC: haematopoietic stem cell
ISCT: international Society for Cellular Therapy
ISEV: international Society for Extracellular Vesicles
SSC: skeletal stem cell
